# Kinetic Values, Mobility (in)equalities, and Ageing in Smart Urban Environments

**DOI:** 10.1007/s10677-021-10249-6

**Published:** 2021-10-28

**Authors:** Jaana Parviainen

**Affiliations:** Faculty of Social Sciences (SOC), Tampere University, Tampere, Finland

**Keywords:** Movement, Kinetic values, The politics of mobility, Ageing urban residents, Urban technological environment

## Abstract

The idea of the right to mobility has been fundamental to modern Western citizenship and is expressed in many legal and government documents. Although there is widespread acceptance regarding the importance of mobility in older adults, there have been few attempts to develop ethical and theoretical tools to portray mobility (in)equalities in old age. This paper develops a novel conceptualisation of kinetic values focusing on older adults whose ability to move has been restricted for internal and external reasons. Informed by the phenomenological theory of kinaesthesia, I suggest that kinetic values are related to four principal dimensions: self-motion, being-moved, co-motion, and forced movement. I assume that these dimensions can address the key dilemma of human dignity among older adults who suffer from losing their autonomy and agency through their mobility impairments and who are at risk of being confined to their homes. To concretise the formulation of kinetic values, I study movement as part of technological equipment and urban infrastructure to examine what kinds of kinetic values mobility services and assistive robots convey for older adults in smart urban environments. By bridging disciplines, the phenomenological approach provides a novel understanding of mobility and the interplay among assistive technologies, kinaesthesia, and urban technological infrastructure. The approach suggests that kinetic values should be interpreted more comprehensively so that kinaesthesia can become better identified as a positive life-promoting practice.

## Introduction

The idea of having the right to mobility has been fundamental to modern Western citizenship and is expressed in many legal and government documents, including the Universal Declaration of Human Rights (UDHR; Blomley 1994; Cresswell 2010). The practice of restricting movement, such as imprisonment, can be seen as a fundamental interference with human rights. Throughout the COVID-19 pandemic, various physical, psychological, and moral effects of restrictions on movement have materialised for many people who previously took their everyday mobility for granted. Still, the right to travel and move freely has been highly uneven across countries, regions, and groups of people within countries. Thus, mobility as a resource is always achievable in different ways for different groups of people. As a right and a resource, mobility can also be understood as a cultural and social value that is pursued for various reasons. For instance, advertising for cars, scooters, mobile phones, and many other products has long appealed to people’s sense of experiencing freedom through movement. When the right to mobility and freedom of movement are wrapped around a commercial or liberal discourse, mobility can become easily naturalised as an individual property (Cresswell 2006). Sheller and Urry (2006) suggest that mobility is not a question of privileging a ‘mobile subjectivity’, but rather of tracking the power of discourses and practices of mobility in creating both movement and stasis. So, how can liberal discourse be avoided when discussing fundamental social and cultural values associated with movement? How can we outline the complexity of kinetic values related to the good life from an ethical and existential perspective?

For the past 20 years, the ‘mobilities paradigm’ in the social sciences and humanities has provided a conceptualisation for scholars to ‘rethink the relation between bodies, movement, and space’ (Sheller 2014). The mobilities approach wants to join a diverse array of forms of movement across scales ranging from the body to the globe (Cresswell 2010, 18). Focusing on movement and mobility and interpreting them as socially and culturally sensitive phenomena, researchers have attempted to account for issues of inequality and social inclusion from the perspective of mobility and immobility (Cresswell 2006; Graham and Marvin 2001; Hidayati, Tan and Yamu 2021; Sheller 2014, 2018). Mobility to promote the careers of young adults (e.g. university exchange-student programmes), control over mobility (e.g. illegal refugees), or prevention of immobility in old age (e.g. sport clubs for seniors) reflect and reinforce power in various ways depending on the group of people they affect. In the case of older adults, a number of empirical studies have demonstrated the health benefits of mobility for older adults (Metz 2000; Ottoni et al. 2016; Rowles 1983; Stalvey et al. 1999) and portrayed various forms of mobility (in)justices in old age (Franke et al. 2020; Webber et al. 2010). Since many of these studies have been related to health sciences or gerontology, the discussion of mobility in old age is closely linked to issues of physical (in)capability and physical movements from the medical point of view, without giving profound consideration to the social dimension in tactile—kinaesthetic feelings that phenomenologists have seen as vital for meaningful human life. Dominant cultural beliefs on ageing easily focus on defining ageing only using health and medical terms. As a paradigm, this ‘medicalisation’ emphasises physiological conditions and biomedical interventions on ageing human bodies (Estes and Binney 1989; Kaufman et al. 2004). The medical-engineering approach to ageing bodies seeks primary solutions for their dilemmas by developing technical solutions or technological devices.

During recent years, many efforts have been made to design new assistive technologies and accessible architectures for older adults to support their physical and mental capabilities so that they can live independently in their homes and move safely outside. In the 2016 report on the European Union’s (EU’s) legislative proposal for the development of robotics, *care robots* for elderly people were seen as a solution, ‘assuming that, when robotics are used to assist people, it will be the family that puts the robot at the disposal of an elderly or disabled person’ (European Parliament 2016, 24). In their literature review, Bedaf et al. (2015) identified about 50 mobility-assisting robot prototypes for the elderly, while only four of these devices were commercially available in 2015. For instance, the Mobiserv robot was one of several prototypes that received research funding from the European Commission in the early 2010 s for developing robotic systems for elderly care (European Commission 2013; Mobiserv Project 2016). The robot is a mobile wheeled semi-humanoid figure equipped with cameras, sensors, sound-system and a touch screen interface. It was designed to remind users to take their medication, do exercises and drink water regularly. As part of a larger smart home environment, one of the tasks of the robot was to monitor users’ vital signs and alert emergency services if needed. A robot as an exercise instructor may seem promising from a physiological perspective, but older adults may feel oppressed by its top-down commands (de Graaf et al. 2017). People may feel they are losing their self-determination over their own movements when they are expected to follow robotic commands. In monitoring the physical body and giving instructions for daily activities, robotic care easily creates feelings of being controlled by a machine at home.

Biomonitoring systems and remote care via mobile devices may provide some vital help for older adults at home, but they rarely alleviate the crippling loneliness of the homebound that results from staying inside for weeks or months. So far, most mobile care robots are not yet safe to be used outside in urban settings (e.g. they are not waterproof, they are limited by battery technology, and they have functional problems in low temperatures). From the beginning of the 2010 s, the concept of the smart city as a new urban policy and strategy has captured the idea of how digitalisation and technologies might lead to the development of innovative solutions for making a city more accessible and alive, provide high-quality services for residents and tourists, and enhance the competitiveness of cities by attracting new investments. However, there are tensions between the visions of smart cities and the available solutions in assisting walking and transitions, especially from the perspectives of ageing residents. No matter how intelligently urban planning is implemented, there are constant challenges for people with reduced mobility; for example, if there are no benches where they can rest after walking 50–100 m, older residents do not dare to walk far from home. In some cities, the incline of the street, uneven pavement, building sites, or fast scooters operating on the sidewalks can cause a fear of falling that will prevent the elderly from leaving home.

In the visions of smart cities, the high-speed train lines that pass from an airport to the city centre, connecting metropolises on a global scale, strongly contrast with the everyday experiences of elderly riders; specifically, some people feel like outsiders when they cannot adjust their bodies to the fast rhythm and automation that are designed primarily for agile young adults. The smart urban infrastructure and its mobility constitute kinetic hierarchies and new boundaries between private and public. In discussing city life from the perspective of ageing residents, these subjects are easily seen as ‘friction’ that stymies progress, making certain people unable to get involved in ‘logistics chains’, thus leaving them outside the public sphere. While slowness, such as the practice of yoga or ‘hygge’, for example, are often seen as desirable values for young city residents, ageing residents who walk too slowly down a pedestrian crossing are not appreciated for their sluggishness. The ideals of speed and slowness vary for different groups, yet it can be argued that limited mobility contributes to exclusion and social isolation in many ways.

In this article, I study ageing from the perspective of the phenomenology of the body; in particular, my approach sheds light on why kinaesthesia and movement need special attention from the ethical point of view in discussing mobility (in)equalities in old age. The theoretical background relies on three main sources: the phenomenological theory of kinaesthesia, critical discussion of (un)equal mobility in the mobilities approach, and technology studies of urban infrastructure and robotics. First, the article explores the politics of mobility to consider how ageing and the consequent increasing restrictions on mobility often lead to growing inequality or social exclusion. Turning to a discussion of autonomy and dignity among older adults, I consider what kind of conceptualisation Nussbaum’s capabilities approach (CA) provides and why phenomenological formulations are needed to understand kinetic values. Next, in defining the notion of kinetic values, I outline four key concepts: self-motion, being-moved, co-motion, and forced movement. I assume that these dimensions can address the key dilemma of human dignity among older adults who suffer from losing their autonomy and agency through their mobility impairments and who are at risk of being confined in their homes. The paper concludes by discussing how kinetic values can be considered in developing mobility services and new technologies for ageing residents in the framework of urban space.

## Ageing, Agency and Dignity

The novel research field of robot ethics in elderly care has arisen from concerns over the effects and impacts of robot care on older adults in the future. Some of this work explores the principles and guidelines of ‘roboethics’ in general (Borenstein and Pearson 2010; Vallor 2016), but most scholars examine issues, such as attentiveness, responsibility, competence, and reciprocity in elderly care (Coeckelbergh 2010; Sharkey and Sharkey 2012; Sparrow and Sparrow 2006; Turkle 2011). In these ethical discussions, there has been little interest in considering how care robots make or do not make adult bodies feel capable of moving or losing control of their own movements, though many new solutions assist the user’s movements. Mobility-assisting robot prototypes are being designed to lift and carry objects, help with toileting and dressing, support eating and drinking, and assist with walking. One of the commercially available robots, the My Spoon Robot, can aid in eating for those who are unable to coordinate their dexterity (Bedaf et al. 2015). Typically, ageing bodies have been approached at the level of physical bodies, and their relations to other bodies and things have been calculated using agent-based movement models to depict patterns of behaviour (Torrens 2016).

In the medical-engineering paradigm, devices are frequently designed in a physiology-driven way. Thus, in care ethics, the issue of dignity has become a central concern in discussing technological solutions for older adults in relation to their social vulnerability and autonomy. Contradictions arise from the tension over how to guarantee the autonomy and agency of the elderly, even if they are dependent on human caregivers, medicine, or assistive technologies. The empirical and theoretical literature relating to the dignity of older people is extensive, drawing upon, in particular, Amartya Sen’s and Martha Nussbaum’s CA (Nussbaum and Sen 1993). Recent discussions of robot ethics in the context of elderly care have also relied on the conceptualisation of CA (Borenstein and Pearson 2010; Vallor 2011).

Agency is taken seriously in CA since capabilities refer to both the potential and actual power of what a person is able to do and achieve in terms of valued choices. The notion of agency is understood here as the capacity of individuals to act independently and to make their own free choices. Developing CA, Nussbaum (2006) identified ten essential functions required for well-being: life; bodily health; bodily integrity; senses, imagination, and thought; emotions; practical reason; affiliations; other species; play; and control over one’s environment. From the perspective of the politics of mobility, Nussbaum’s (2006) moral concept of bodily integrity emphasises the importance of personal autonomy, the right to move freely, and self-determination over one’s physical body. Nussbaum’s view of bodily integrity is based on a liberal way of conceptualising subjectivity in which the mind and the body are separate, so bodily integrity refers to physical inviolability (Patosalmi 2009). Nussbaum’s view of bodily integrity presents important baselines for body politics that contributes to many invaluable human rights protections, including the freedom to move freely or the identification of sexual integrity, but it nevertheless marshals a highly abstract, disembodied, idealised, and anaemic vision of human mobility and movement (Parviainen and Pirhonen 2017). Having been developed as an approach to global development and justice, the conceptualisation of bodily integrity remains in a dualistic trap when it emphasises the difference between the physical and psychological dimensions of human existence. Nussbaum’s ‘list of capability’ has also been criticised for being firmly rooted in Aristotelian ideals, being paternalistic, and representing the views of middle-class Western subjectivities (Mcnaughton Nicholls 2010; Stewart 2001).

Reaching beyond a dualistic discourse of adult individuals, Pia Kontos (2005, 558) proposes that the ageing body and its capabilities should be seen as an ‘active, communicative agent, imbued with its own wisdom, intentionality, and purposefulness’. Following Maurice Merleau-Ponty’s (1989) phenomenological conceptualisation, the lived body is understood as the lived centre of consciousness and is often associated with selfhood.^2^ In the distinction between the lived body and the physical body, the notion of the lived body captures the body in the first-person perspective, whereas the physical body is a material entity. Furthermore, relying on Husserl’s phenomenology, Merleau-Ponty (1989, 137) addresses the notion of motor intentionality, which concerns the lived body’s intention towards objects, its directing itself towards goals, and its acting in a way that enables it to make sense of a collection of disparate bodily movements, unifying them into meaningful action. In this sense, the notion of ‘physical movement’ is inadequate to prescribe how lived bodies share their lives and intimacy with other living beings through meaningful gestures, movement, and touching. Therefore, phenomenological analytical methods can provide appropriate conceptual tools to identify kinetic values in human life that open up experiential perspectives into movement.

In the early 20^th^ century, interest in the study of movement experience, so-called ‘kinaesthesia’, sparked the phenomenological tradition, along with Edmund Husserl’s (1973) and Edith Stein’s (1989) studies. According to Husserl (1973), kinaesthesia makes it possible for lived bodies to appraise material and spatial features in an environment what mere visual or verbal information cannot reveal. Through kinaesthetic sensation and movement, lived bodies attain a real understanding of the three-dimensional and material world. By bodily movements, lived bodies tend to evaluate the characteristics of inanimate beings by coming near, walking around, touching them and stepping back to set the right perspective to better understand them. Husserl argues that the kinaesthetic sense and spatial movement have a central organising role for perceptions as a whole.

More recently, discussions by phenomenologists (Sheets-Johnstone 1999) and philosophers in the cognitive sciences (Gibbs 2005) have suggested that philosophical analysis should pay more attention to the role and relevance of movement in perceptual systems and cognition. For Sheets-Johnstone (1999; 2010), the kinaesthetic sense is something that helps us recognise differences and similarities in our own movement qualities and haptic sensations but also identify objects and their movements around us. For instance, when we lift a suitcase, this movement reveals something about its weight. Swiping the suitcase with our hand tells us about its texture and shape. Sitting on top of a suitcase says something about its rigidity and compressibility. Thus, bodily movements appear fundamentally intentional and ‘mindful’ in the way that they entail a special kind of reflective thinking. Kinaesthesia can be rigorous and mindful and without being conceptual and verbal (Parviainen and Aromaa 2017).

On the basis of this discussion, I argue that kinaesthesia has a crucial role in how bodies communicate through gestures, anticipate what others intend to do, and receive information from our environment through their movements. Focusing on kinaesthesia in interpreting socially, politically, and culturally sensitive phenomena, it is important to account for issues of inequality or social exclusion from the perspectives of kinetic values. Mobility has been seen as a resource and a privilege to which not everyone in society has an equal relationship. The politics of mobility should not only be about a ‘quantitative’ resource but also outline the ‘qualitative’ features of the movement that impact how people as mobile agents can live the good life.

## A Phenomenological Account of Kinetic Values

Formulating ‘kinetic values’ from the perspective of ageing residents, it is essential to understand the moving agent’s ownership of movement—who or what produces the movement and who feels it as their own. Gallagher (2000) and Burin et al. (2015) suggest that bodily movements have a significant role in developing and maintaining a coherent body ownership, which is closely related to bodily integrity. Bodily movements and the sense of body ownership are inherently connected, so that is why lived bodies become aware of self-generated movements compared to bodily movements that are generated by other beings (Gallagher 2000). By ‘self-motion’ I refer to self-generated movements when lived bodies recognise their agency by intentionally moving and feeling their movements physically, affectively, or socially resonating with the external world.

As sated above, lived bodies not only move themselves, but also recognise situations in which they are ‘moved’ by other beings (i.e. ‘movers’). For instance, this ‘being-moved’ occurs when someone bumps into me accidentally on the street and I lose my balance. Here, I recognise the rapid transition from being-moving to self-motion when reaching my balance again. Being-moved may include varying degrees of embodied consent, including complete surrender to the device being taken (such as taking a subway ride) or reluctant dragging (such as of a dog on a leash). Some functions of assisting mobile robots also fall into this category, such as lifting robots, although these are not yet on the market. Following Stein’s (1989) formulation of movement categories, we can make at least three kinds of distinctions as to what kind of intentional act the mover is performing. The ontological nature of the mover is of great importance, as is what kind of movement it produces. For Stein, ‘alive movement’ is voluntary, active, and unpredictable; the mover is kinetically aware of their own movement, much like the person who bumps me accidentally on the street or a dog walker who pulls a dog. ‘Mechanical movement’ is passive and predictable, such as the motion produced by a lifting robot for a patient or a metro for passengers. ‘Spontaneous movement’, in turn, is involuntary, uncontrollable, and unpredictable, but it is not alive: the mover is not aware of its own movement (such as a tree falling in a storm).

Stein’s reflection on motion is somewhat reminiscent of the ways that pre-Socratic philosophers and Aristotle viewed motion in their natural philosophies. Aristotle made the distinction between self-motion and being-moved when he was considering why some things are produced spontaneously while others are not. People are able to move themselves, which, according to Aristotle, means that the mover and the movement are together. Animals are also able to move themselves, but very few, mostly humans, can move themselves in a particular way, such as dancing. In discussing the conditions of being-moved, Aristotle says, ‘The things, then, whose matter is of this sort, e.g. stones, cannot be moved in the particular way required, except by something else, but in another way they can move themselves—and so it is with fire’ (*Met* 7.9, 1034a15–18). He suggests that stones cannot move by themselves but need some other sources to be moved.

Stein’s and Aristotle’s reflections on movement may seem irrelevant when it comes to mobility services, kinaesthesia, and smart cities, but I dare to suggest two more new categories of movement that can clarify kinaesthetic experience and movement ownership from the perspective of the politics of mobility in the socio-technically driven world. These two categories of movement address positive and negative aspects of losing ownership over one’s movement. The third category added is when the agent organises their own bodily movements together with some other being. Kinaesthetic feelings have a key role in self-identification, which in turn makes it possible to build and evaluate relationships with other people. Stepping away or moving closer are ways to form relationships with others. Feeling ‘co-motion’ with the other being, for example, is what happens when the agent is walking next to someone at the same pace. Similarly, birds flying in formations support their co-motion to adjust their energy in their regular seasonal migration. In these cases, agents are moving intentionally without being forced by some dominant agent or external pressure.

Co-motion differs from the fourth category, ‘forced movement’; this is conducted, for instance, in a military parade, where formations of soldiers have their movement restricted by close-order manoeuvring, such as marching. In the case of forced movement, the system and its pace move their bodies even if they may sometimes mistakenly think that their movements are intentionally self-motion. It should be remembered that, in some cases, forced movement (holding) can protect the agent from harming themselves. For this reason, it must also be called a kinetic value in the positive sense. Lefebvre (2004) has captured a difference between co-motion and forced movement by discussing rhythm. In Lefebvre’s terms, the notion of forced movement can be understood when the exterior rhythm of rationalised time and space comes into contradiction with lived and embodied rhythm (Lefebvre 2004, 9).

**Figure 1:**
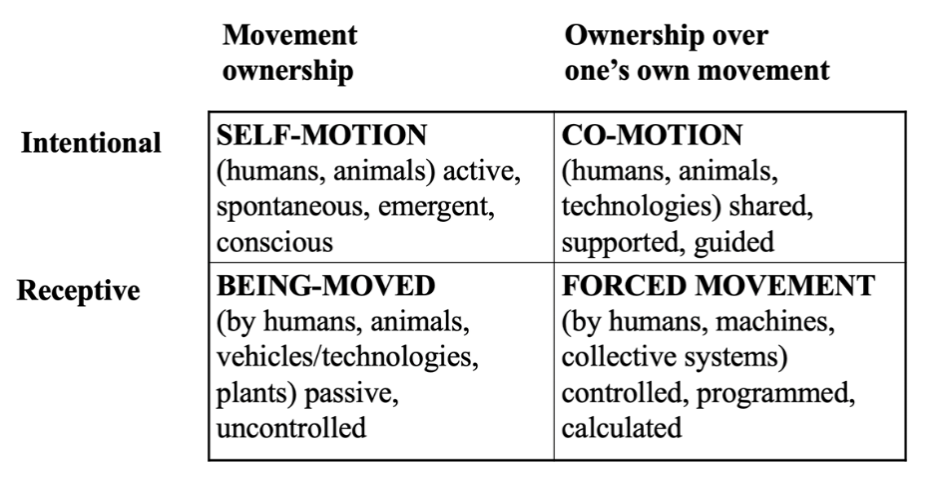
Kinetic values related to four dimensions of movement

I suggest that these four aspects of movement—self-motion, being-moved, co-motion, and forced movement—constitute a central framework for kinetic values, which can be used to examine the significance of movement in human life (Fig. 1). Kinetic values refer to the various dimensions of freedom to move, including that individuals have the right to self-motion and co-motion with others but they do not need be involved in being-moved or forced movement against their moral principles (Parviainen & Särkikoski 2018). This is particularly important in the case of older adults, who are increasingly confronted with situations where they are moved by technological devices. By relying only on the concept of bodily integrity, we cannot identify characteristics related to whether the movement of a technical device evokes comfortable and motivating or patronizing and humiliating feelings. By identifying the kinaesthetic differences between these four types of movements, we can analyse what kind of morally problematic situations technological devices can cause although these devices are approved for health safety.

To recognise kinetic values, we must also take into account the spatial dimensions of movement. Although proprioception has been associated with kinesthesia, it is more essential to identify the trajectories of space to distinguish two levels of motion, microlevel and local-level movements (Parviainen 2016). By ‘microlevel movements’ I mean fine motor actions, such as hand-eye coordination, or internal somatic movements, such as breathing and postural control. ‘Microlevel movements’ take place within one’s kinesphere that refers to the intimate lived space, reached easily by extended limbs (Laban 1966). Ownership experiences related to one’s own kinesphere are crucial when people feel that their intimate space is invaded without their own permission. Intrusion does not mean not only acts of physical violence or sexual harassment, but also negative affections such as condescension and ignoring another person’s embodied presence. Negative affections can be felt as living in a hostile and discouraging atmosphere that freezes people’s action and prevents them from carrying out their potential. In such a situation, people feel paralyzed in their kinesphere without the ability to develop co-motion and share their kinesphere with others. So, a kinesphere can easily feel like a prison if one’s own movements are restricted to the personal space. This may also explain why symptoms of depression and anxiety have been observed to increase with COVID-19 movement restrictions (Abdul Khaiyom 2020). In this sense, by developing the conceptualisation of kinetic values, we can address how a hostile environment or immobility can be detrimental to individuals even if their bodily integrity is not threatened.

Lived bodies always carry their kinesphere with them wherever they go but ‘local-level movements’ concern movement trajectories that they perform in reaching neighbourhood facilities or participating in meaningful social, cultural, and physical activities ‘out-of-home’. These movement trajectories constitute spatial choreographies that lived bodies make in social interaction with other living beings and material settings, such as walking on the street. Stalvey and his colleagues shed light on this dimension of movement by defining it as ‘the spatial extent of one’s travel within the environment’, encompassing ‘travel in, around, and outside the home as one conducts the business and social aspects of everyday life’ (Stalvey et al. 1999, 461). From the perspective of kinetic values, to make such choreographies is not just the basic human right to move freely; it also concerns the right to feel through kinaesthesia other living beings and material settings outside their residences. Thus, choreographies are not instruments to achieve anything but the right to have spatial self-motion that has a physical or social influence on the external world.

The kinetic values described above make it possible to assess the extent to which the services provided to older adults and designed robotics offer opportunities for them to sustain their self-motion and build shared movement with others, and to what extent they are merely being-moved or forced into movement. In old age, progressing diseases and the consequent impairments and functional limitations reduce the mobility of bodies, but it is important to stress here that the embodied capabilities that distinguish the four dimensions of kinetic values do not deteriorate or disappear with ageing. I continue my discussion of how kinetic values are considered in developing the assistive technologies and other well-being services for elderly people in the framework of urban environments.

## Ageing in Urban Technological Infrastructure

Many recent empirical studies show that the isolation and loneliness of older people can be alleviated through remote connections, video calls, and the use of social media, but technological solutions do not replace in-person and outer-household contacts (Brooke and Clark 2020; Hu and Qian 2021)—in other words, local-level spatial choreographies outside the home. Restrictions on mobility, whether related to external constraints (e.g. policies implemented during the COVID-19 pandemic) or a person’s own mobility limitations, are linked to loneliness and social isolation, easily forming a vicious circle. When an older adult with mobility limitations lacks resources to overcome many of the constraints imposed by their immediate surroundings, this leads gradually to the individual’s ability to develop and maintain social relationships and ultimately to loneliness and social isolation. Here loneliness is defined as a subjective feeling of anxiety and dissatisfaction with connectedness with others and a lack of the desired quality and quantity of social relationships (Victor et al. 2005). Of course, not all isolation experiences (i.e. mediation retreats) cause anxiety, but can instead be relieving. From the perspective of kinetic values, feelings of being imprisoned in one’s own apartment can be understood as the experience of a forced movement of stillness, causing feelings of being trapped in one’s head (mind) with feelings of anxiety and depression. The experience of a forced movement can be related to feelings of the lack of body ownership, feelings of loss of agency, disembodiment feelings, and feelings of becoming ‘invisible’ to others. I hypothesise here that close and dynamic physical and synchronous interactions with the environment could make older people feel more present in their bodies and less ‘trapped’ in their minds.

In countries where the proportion of older people is growing, new inclusive policies, services, and programs have been developed for supporting older people’s mobility, from special transportation services regarding everyday shopping to well-equipped rehabilitation centres. In this context, many new vehicles, such as robotic walkers (Lee et al. 2010), autonomous mobility scooters (Ntaki et al. 2012), and exoskeletons as wearable technologies (Bock et al. 2012), are being developed to assist ageing pedestrians in their movement trajectories in urban settings. Typically, the design ideals behind the many prototypes of assistive robotics rely on liberal discourse in which mobility is naturalized as an individual property closely engaged with individual bodies as mobile subjects. In the case of exoskeletons, for instance, robot actuators ideally support an individual body’s movements, blurring the boundary between human movement and the technological device and forming a feeling of co-motion. Although American and Japanese robotics scientists have been experimenting with complete mobile costumes and power-assistance devices since the 1970 s, development projects have not been successful enough to provide comfortable wearable robotics at an affordable price to consumers. Providing comfortable feelings of co-movement between exoskeletons and human movements has remained unresolved for a number of reasons, including security issues. For example, sudden unintentional or unplanned movements can cause a nasty experience if the device is unable to react smoothly to the user’s movements. Designing exoskeletons to support co-motion with the human body does not depend solely on the knowledge of biomechanics and complex neuromuscular systems, but also studying how exoskeletons respond to the kinesthetic feelings of self-esteem of individual bodies when this type of technology is used. If the kinesthetic feelings of self-movement and co-movement with these techniques are not taken seriously, users may end up feeling that powerful machines are forcing them to move in humiliating ways.

The approach of kinetic values can pave the way for studying how embodied adaptation to assistive technologies interrelates with digitalized urban structures, as well as how, among other things, the policies of city planning are affected and challenged in the process. Electric scooters are a common sight, changing radically the dynamism of traffic on many streets in European and North American cities. These scooters have become an increasingly popular vehicle among young adults, providing an experience of an urban sense of freedom: the wind flutters through the driver’s hair as the e-scooter curves smoothly along the sidewalk. Still, it is older adults who are in dire need of affordable and easily accessible equipment that would make it easy to move around the city. Safe vehicles for the elderly would open up new opportunities for older residents to get to the city centre, go shopping, visit friends, and walk in parks—not to mention producing a new kind of kinetic experience of freedom through movement. However, it is unlikely that global equipment manufacturers will see older adults as potential clients for whom they can provide safe assistive transportation, even as the ageing group grows as consumers at an accelerating rate. It seems that without the significant contribution of the state, municipalities, and the third sector, the problems of inequality in urban mobility for the elderly will not be solved.

So, how can new services and technologies for older adults be designed in such a manner that ageing residents who suffer from physical limitations can sustain their dignity through opportunities for safely choosing their movement trajectory in an urban environment? How can they be offered kinetic experiences in which they feel part of the urban kinaesthetic field and not separated or detached from it? One of the basic kinetic values formulated above—feeling co-motion with other beings—has been promoted in the project ‘Cycling Without Age’ (CWA). In this service concept, volunteers (pedalling pilots) transport the elderly and other people with reduced mobility on rickshaws (CWA 2021). In special bicycle taxis for one pilot (back) and two passengers (front), the pedalling pilots share their physical power of movement with the passengers by generating a feeling of co-motion. Passengers recognise that they are being moved, but they may feel involved in the joint movement as they feel kinaesthetic empathy for the physical work of the pilot. Being seated close together in the rickshaw provides a kinesphere in which the intimate lived space can be shared between the pilot and the passengers. By combining slow rides with talking with passengers, rickshaw rides attempt to build new relationships between generations, among older adults, between pilots and passengers, and among care home employees and family members. Slow cycling allows passengers to sense the environment and be present in the moment, letting ageing residents share their memories and experiences related to places they pass on the ride. In addition, as an ecological way to transport someone, rickshaw rides provide for ageing residents the right to experience the city and nature close up from the bicycle. Although activists in the CWA project do not talk about kinetic values explicitly, the central principals of the service concept meet some basic ideas of kinetic values: feelings of body ownership by choosing comfortable movement trajectories, feelings of co-motion and a shared kinesphere with others, having a connection to the environment through movement, and an experience of freedom though motion, particularly by ‘feel[ing] the wind through their hair again’ (CWA 2021).

Service concepts such as CWA are not the sole answer to many of the severe problems of mobility inequalities for ageing residents in cities. However, they show that mobility is not a technical transition from place A to place B, but involves many dimensions that are valuable to achieving the good life that one usually recognises along intrinsic or extrinsic mobility restrictions. Through an understanding of the principles of kinetic values, it is possible to outline how the interplay between technologies, kinaesthesia, and urban infrastructure works at large. Developing mobility solutions for ageing residents, politicians and city planners should also re-think what kind of kinetic values they provide through their services. Instead of starting to invest resources in developing ‘top-down’ technological applications as solutions for social problems in ageing, more could be done to encourage a range of ‘bottom-up’ services for ageing residents, including new mobility services. The most severe problems of ageing are not necessarily medical problems but economic and social ones, namely, poverty, social isolation, and loneliness. Linking the discussion on older people to their medical problems, including possible robotic solutions, the more relevant question is: How could smart cities be developed to be more vital and culturally and economically attractive, not just to ageing residents but for all? Identifying kinetic values in urban planning could open up a new perspective on how the cities of the future could be designed in a way that considers experientiality combined with social equality.

## Conclusions

This article was motivated by the observation that many valuable aspects of the good life are made possible by movement; at the same time, many people with reduced mobility are left out of these experiences. One of my leading questions was how to outline fundamental but complex social and cultural values associated with movement. I argued that the previous ethical conceptualisations of bodily integrity and discussions of right to mobility do not provide a sufficient theoretical basis when speaking of mobility (in)equalities in old age. The conceptualisation of bodily integrity remains in a dualistic trap when emphasising the difference between physical movement and mental states. Turning to recent debates on the politics of mobility and phenomenological discussions of kinaesthesia, I proposed that movement has a crucial role in self-identification and building affective relationships with other people, while (im)mobility also makes it possible to constitute kinetic hierarchies in society. From the perspective of ageing residents, internal or external movement constraints, in particular, are associated with symptoms of depression, loneliness, and social isolation.

Applying the phenomenological theory of kinaesthesia, the four principal dimensions of kinetic values were outlined—self-motion, being-moved, co-motion, and forced movement—with emphasis placed on the idea that movement is inherently a relational and spatial phenomenon. I suggested that these kinetic dimensions are fundamental when assessing how the autonomy and dignity of older adults are identified in the design of environments, services, or technological devices. Using examples of new mobility services and movement-assisting robot prototypes, this discussion addressed that new devices and services, found to be functional, helpful, and safe in the research context of the medical-engineering paradigm, can still be considered patronising and humiliating when they are considered from the perspective of kinetic values. Human-facing biomonitoring robotics can be easily felt as a control system if people feel that they are losing their self-determination over their movements. Similarly, robotic walkers that aim at supporting human mobility can feel humiliating and patronising when their external power forces one to change their habitual walking style.

In the last section of the article, describing how the CWA mobility concept has promoted kinetic values, I argued that new services and technologies for ageing residents can also be designed in such manner that the people who suffer from physical limitations can sustain their dignity by providing feelings of co-motion with other people. The CWA concept showed that older adults can be offered kinetic experiences in which they feel part of the urban kinaesthetic field and not separated or detached from it. Still, reading the CWA concept and other examples from the kinetic perspective is hampered by the fact that very little empirical research, if any, has been done around kinetic values. In this respect, there is still much ground to be covered to better understand how to foster the theory of kinetic values and apply its concepts in empirical research. My proposed directions for future research represent some of the ways that researchers can move forward within this line of inquiry.

## Data Availability

Not applicable.

## Footnotes

2 More exactly, Merleau-Ponty (1989) states that ‘[c]onsciousness is being-towards-the-thing through the intermediary of the body’ (138–139).
